# Formation of Neural Precursor Cell Populations by Differentiation of Embryonic Stem Cells In Vitro

**DOI:** 10.1100/tsw.2002.134

**Published:** 2002-03-12

**Authors:** Joy Rathjen, Peter D. Rathjen

**Affiliations:** Department of Molecular Biosciences, Adelaide University, Australia; ARC SRC for Molecular Genetics of Development, Adelaide University, Australia

**Keywords:** ES cells, differentiation, neural lineages, neurectoderm, neural epithelium, retinoic acid (RA), nestin

## Abstract

Recent interest in the generation of neural lineages by differentiation of embryonic stem cells arises from the opportunities represented by a developmentally normal, unlimited source of material that can be manipulated genetically with precision. Several experimental approaches, which differ conceptually, in the route of differentiation and the characteristics of the resulting cell population have been reported. In this review we undertake a comparative analysis of these approaches and their suitability for experimental investigation or implantation.

## INTRODUCTION

Potential exploitations of the pluripotent differentiation capability of embryonic stem (ES) cells have long been recognised[1]. Investigations have gained recent momentum following reports of the isolation of human ES cells[2,3], bringing closer the possibility of harnessing ES cell differentiation for the production of differentiated cells with therapeutic potential. Increasingly, too, ES cells are being used as a tool for characterising at cellular and molecular level processes of cell differentiation and fate specification[1]. ES cells bring several technical advantages to these endeavours. As an immortal, self-renewing population they represent an unlimited supply of starting material, with a broad developmental potential that reflects their origins from the Inner Cell Mass (ICM) founder population of the mammalian embryo. Further, the ability to introduce into ES cells precision genomic modifications by homologous recombination provides an opportunity to couple the power of ES cell biology with genetic analysis for exploration of the molecular basis of cell function and development.

The generation of neural lineages from ES cells has received considerable attention, both because of fundamental interest in the establishment and patterning of these lineages during embryogenesis, and because the immunoprivileged neural environment *in vivo* makes such cell types prospective early candidates for therapeutic implantation to treat neurodegenerative conditions such as Parkinson's disease and diseases caused by cell loss, e.g., stroke and spinal cord injury. Experiments in laboratory animal models and, in the case of Parkinson disease, in affected individuals, provide potential proof of concept of the use of such cell replacement therapies[4,5,6,51]. The generation of neural precursors, which can further differentiate to neural cell types *in vitro* or *in vivo* in response to endogenous signalling is an important first step in realising this potential.–

Neural fate determination in the embryo occurs via a temporally and spatially regulated process that results in the progressive elaboration of successive cell populations from the ICM. Differentiation to a second pluripotent cell population, primitive ectoderm is followed, during gastrulation, by sequential formation of the ectodermal germ layer and neurectoderm. Originally seen as a sheet of cells along the anterior midline of the embryo, the neural plate folds and joins to give rise to the neural tube. The multipotent precursor cells comprising the neural plate/tube differentiate to the major cell types of the central nervous system (CNS), neurons, glia, and the peripheral nervous system (PNS) through formation of the neural crest lineage. In this review we assess recent progress in the formation of neural precursors from pluripotent ES cells with particular consideration of the relationship between these cells and those arising during normal embryogenesis. A comprehensive overview of the generation and characterisation of more differentiated neural cell populations can be found in a recent review by Guan et al.[7].

## ES CELLS CAN BE DIFFERENTIATED TO NEURAL LINEAGES

The ability of ES cells to contribute functionally to all tissues of the embryo and the adult, including both the CNS and PNS, after reintroduction into host embryos demonstrates the pluripotent developmental potential of these cells. Early assessment of ES cell potency outside the embryonic environment was achieved through the formation of teratocarcinomas by injection of cells at ectopic sites in the mouse and by formation of embryoid bodies (EBs) *in vitro*[8,9,10]. Unlike teratocarcinomas, in which differentiation is chaotic, differentiation within EBs results in the relatively ordered and predictable elaboration of cell populations in a manner that reiterates embryogenesis, with initial establishment of extraembryonic endoderm and primitive ectoderm populations followed by differentiation to cell populations representative of all three germ layers. Although differentiation within EBs appears to recapitulate early embryonic events, these structures lack the organisation inherent to the developing embryo, with no anterior/posterior or dorsal/ventral specification. Differentiation within these complex environments results in the formation of a wide range of cell types, including beating cardiocytes, red blood cells, bone, cartilage, and neurons[8,10,11,12]. Although formation of the neural lineages has not been analysed in detail, lineage formation within EBs has generally been shown to recapitulate the events of embryogenesis with temporal progression between intermediate cell populations of progressively restricted developmental potential[10,13,14].

While these early reports establish the concept that pluripotent cell differentiation could be used to investigate the processes of neural specification, and to produce neural cell types, exploitation of teratocarcinomas or unmodified EBs for these purposes has been restricted by inherent limitations in these differentiation systems. Neurons comprise only a small fraction of the cells produced within a teratocarcinoma or differentiating EB[15], and differentiation of neural cell types is not temporally synchronous or spatially organised. This compromises the purification of intermediate populations and analysis of differentiation pathways. Endogenous signalling inherent within these complex differentiation environments, arising from visceral endoderm[16] and/or other, non-neural cell populations[1], provides additional complications as

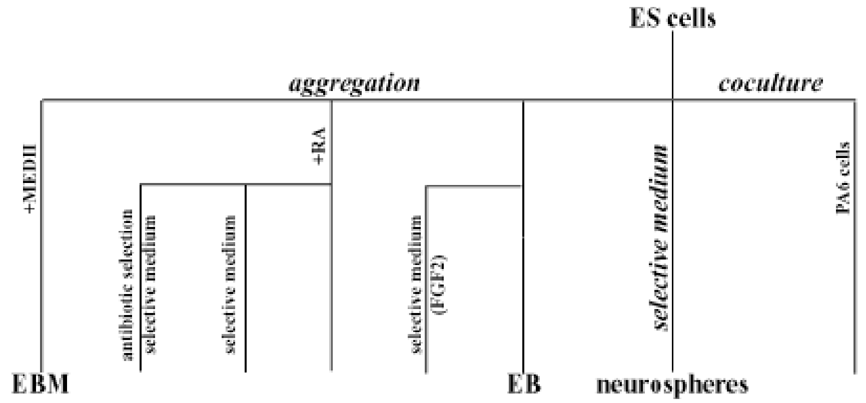

| | | | | | | | | | |
|---|---|---|---|---|---|---|---|---|---|
| neural precursor markers | %nestin$^+$ | present | 94% | 90% | 70% | 85% | present | present | present |
| | %N-CAM$^+$ | 95.7% | ? | ? | ? | ? | ? | ? | (92% colonies) |
| | % Sox1$^+$ | >95% | 89% | 47% | ? | ? | ? | ? | ? |
| differentiation potential | neurons | + | + | + | + | + | + | + | + |
| | glia | + | + | + | + | + | + | + | + |
| | neural crest | + | ? | ? | ? | ? | ? | ? | ? |
| contaminating cell populations | extraembryonic endoderm | not present | ? | ? | ? | ? | + | + | ? |
| | embryonic cell populations | <2% aggregates | + | + | + | + | + | + | <2% colonies |

**FIGURE 1.** Summary of the approaches and outcomes of differentiation of ES cells to neural lineages. The data presented were compiled from[24,31,45,48,50]. Where the data have not been determined, no values have been entered in the table.

differentiating cells are predicted to be exposed to both appropriate and potentially inappropriate patterning and differentiation signals. The deregulated signalling environment precludes stabilisation, expansion, and purification of developmentally labile precursor cell populations and restricts the potential for enforcement of directed differentiation by application of exogenous signals.

Researchers have attempted to overcome such limitations by developing technologies that enrich for the formation of neural cell lineages during pluripotent cell differentiation. Although it is difficult to categorise these approaches as they overlap extensively (Fig. 1), for the purposes of this review the technologies have been grouped into several broad areas—chemical induction of differentiation, spontaneous or chemically induced differentiation combined with selection for neural precursors, and directed ES cell differentiation by coculture of ES cells with inductive cell lines or conditioned medium. All of these methods result in the production of neural precursors; comparative analysis of the gene expression profiles or developmental potential of these cells has not been undertaken. Accordingly, the relationship between these cell populations and their relevance to populations produced during neurogenesis is difficult to ascertain.

## CHEMICAL INDUCTION OF NEURAL CELL LINEAGES FROM ES CELLS: USE OF RETINOIC ACID

Potential roles for retinioc acid in embryonic and postembryonic development, and particularly in the differentiation of cells in the embryonic nervous system, have long been recognised[17,18]. The differentiation of pluripotent cells to neural lineages in response to retinoic acid (RA) was first demonstrated using embryonal carcinoma (EC) cells[19,20]. Subsequent experiments using ES cells demonstrated a similar neural-inducing capability, with effective induction of neural cell populations from aggregated ES cells by addition of all-*trans* RA, at concentrations between $10^{-6}$ and $10^{-7}$ M, to the culture medium[15,21,22]. Neural induction has been observed after addition of RA to EBs during distinct windows of time, for the initial 2 or 4 days of differentiation[15,22,23] or between days 4 and 8 of differentiation[21], suggesting that cells competent to respond to RA induction are present within EBs for at least 4 days. The target cells and the efficiency of RA induction are difficult to address as RA is cytotoxic and induction is accompanied by extensive cell death[24]; however, approximately 70% of surviving cells exhibit neural or glial specific properties after dissociation and plating in serum-free medium[24]. This represents a potent induction of neural lineages when compared with spontaneous differentiation of ES cells[15,21]. Contaminating populations including epithelioid cells and extraembryonic visceral endoderm have been reported[6,24].

Analysis of ES cell aggregates differentiated as EBs for 4 days prior to a further 4-day culture in the presence of $10^{-6}$ M RA demonstrated that formation of neural progenitor cells, identified by expression of the early neuroepithelium markers *Sox1*, and *Sox2*, and nestin, preceded formation of more differentiated neural cell populations[24]. Neural progenitors formed in response to RA have the potential to differentiate to cells of both the neural and glial lineages[22,23,24], consistent with the properties of neuroepithelium *in vivo*, although homogeneous formation of these lineages from RA-induced neural precursors has not been described.

Neurons differentiated from RA-induced neural progenitors appear functionally competent and acquire electrophysical and immunocytochemical properties characteristic of postmitotic nerve cells[15,21,22]. Furthermore, RA-differentiated ES cells implanted into sites of experimentally induced spinal cord injury have been shown to promote some phenotypic recovery[5,6], suggesting that these cells can integrate, survive, and function *in vivo*, although the extent and the efficiency of these processes has yet to be addressed.

Some debate exists as to the developmental potency of neural progenitors induced from pluripotent cells in response to RA. Exposure of mouse embryos to excess RA *in utero* results in posteriorisation of the anterior hindbrain, possibly resulting from misexpression of *Hox* genes, suggesting a relationship between RA concentration, the expression of *Hox* genes and neural cell fate[25,26,27]. Therefore, the possibility exists that induction of differentiation by RA may result in precursor cell populations with limited differentiation potential, or alternatively the use of varying RA concentrations could result in the formation of precursors with different *HOX* gene expression and differentiation potentials. Characterisation of differentiation products induced by RA showed an under-representation of neural subpopulations characteristic of the dorsal CNS and PNS[26]. In contrast, Li et al.[24] demonstrated expression of both the dorsal marker gene *Pax3* and the ventral marker gene *Pax6* within neural precursor cells after induction with RA, suggesting the formation of a heterogeneous neural progenitor population containing cells with both dorsal and ventral characteristics.

The up regulation of genes responsive to positional information, such as *Pax3* and *Pax6*, in neural precursor cells formed by induction with RA suggests the activation of positional signalling pathways within the differentiation environment. Establishment of dorsoventral polarity within the neural tube during embryogenesis occurs in part in response to signals from non-neural cell populations, for instance in response to Shh from the adjacent notochord (ventral specification[29]) and signals arising from the overlying ectoderm (dorsal specification[30]). While the cellular origins of signals involved in patterning of neural precursors during RA induction have not been identified, the presence of patterning presumably reflects the reported cellular heterogeneity within the differentiation environment. This cellular heterogeneity may also underlie the apparently conflicting reports of neural precursor potency[24,28]. The cellular complexity of the differentiation environment and activation of positional signalling pathways suggests that formation of homogeneous neural progenitor populations and enforcement of further homogeneous differentiation in response to exogenous factors is unlikely to be achieved in this system.

## SELECTIVE PURIFICATION OF NEURAL CELL POPULATIONS FROM COMPLEX DIFFERENTIATION ENVIRONMENTS

Selective methods for purification and expansion of the neural precursor population rely on initial establishment of the ectodermal lineage during spontaneous EB differentiation or in response to RA induction, followed by selective procedures that enrich for neural precursors. Selective culture conditions exploit the preferential survival and proliferation of neural precursors in chemically defined medium without serum supplementation[31,32,33,34]. ES cells were aggregated and differentiated as EBs for 4 days prior to plating. Addition of selective medium, a serum-free medium, containing insulin, transferrin, sodium selenite, and fibronectin, on day 5 resulted in death and detachment of a large proportion of the cells, presumably reflecting failure of many non-neural cells to survive in the absence of serum. Of the surviving cells, a large proportion exhibited a neuroepithelial precursor morphology and around 85% expressed nestin[31,34,35]. Addition of FGF2 resulted in increased proliferation of the neural precursor population. While the identity of the non-neural cells has not been characterised extensively, foci of SSEA1 expressing cells suggest the presence of residual pluripotent cells, while keratin 8 immunoreactivity is consistent with survival of a surface ectodermal population[31]. This is perhaps expected as both the surface ectodermal and neural lineages are proposed to share a common ectodermal precursor[36].

More effective neural cell selection has been accomplished using ES cells engineered to express the neomycin resistance gene under the control of the neural specific promoter Sox2[24]. RA induction of differentiation on days 4 to 8 followed by dissociation and culture in selective medium in the presence of G418 resulted in cell populations comprising >90% nestin positive neural progenitor cells, and coexpressing the early neural markers Sox1 and Sox2. A similar approach using ES cells expressing the neomycin resistance gene under the control of the neural specific enhancer of the nestin gene has been reported[37], although characterisation of the cells produced by this methodology is preliminary.

The neural progenitor cell populations obtained by selection of neural precursors from EBs have been identified as equivalent to undifferentiated neural epithelium by expression of nestin[31,32,33,34,35] and the brain fatty acid protein[31]. Consistent with this identification, the cells have the ability to differentiate into both neurons and glia *in vitro*, although formation of neural crest has not been demonstrated. After implantation of these cells to the telencephalic vesicles of 16.5 to 18.5 d.p.c. rat embryos *in utero*, contribution of donor cells could be observed in a number of host brain regions, demonstrating both survival and dispersal within the brain. Furthermore, donor cells had integrated into the host tissue and differentiated into neurons, astrocytes, and oligodendrocytes, demonstrating a developmental potential consistent with early neural precursors[38]. Implantation of similar ES cell–derived neural progenitors into the adult rat brain striatum also demonstrated survival, spreading, integration, and differentiation to neurons and glial cells[35].

In contrast to spontaneous and RA-induced differentiation protocols, the use of selective methods allows proliferation of populations enriched in undifferentiated neural precursor cells, with limited spontaneous differentiation to terminally differentiated cells. This possibly reflects selective loss of contaminating cell populations and, consequentially, the loss of differentiation-inducing signals within the differentiation environment. These relatively stable, proliferating neural precursors have been used as substrates for further, directed differentiation into populations enriched in specific neural lineages. Sequential addition of FGF2, FGF2 + EGF, and PDGF resulted in an isomorphous population of cells immunoreactive to the glial precursor marker A2B5. Subsequent withdrawal of growth factors led to the appearance within the culture of both oligodendrocytes (38.3%) and astrocytes (35.7%), identified by immunoreactivity to O4 and expression of GFAP respectively[32]. The identity of other cells within the population was not reported. Differentiation of precursors in medium supplemented with sonic hedgehog, FGF8, db-cAMP, and ascorbic acid, or "survival promoting factors" (IL-1β, GDNF, NTN, TGF-$\beta_3$, and db-cAMP), resulted in enrichment for dopaminergic neurons, with 34 and 43% of the resulting neurons positive for tyrosine hydroxylase (TH) expression, respectively[33,35].

Neurons formed by further differentiation of these precursors *in vitro* exhibit electrophysiological characteristics consistent with postmitotic neurons[31,32] and have been demonstrated to secrete neurotransmitters[33,34]. Further, cell populations enriched in glial precursors, formed as described above and implanted into the dorsal columns of myelin deficient (md) rat spinal cords, showed survival, integration with host tissues, and differentiation to functional myelinating oligodendrocytes and astrocytes after 2 weeks[32].

The acquisition of positional specification within neural progenitors generated from ES cells by selective techniques has not been studied extensively, precluding a full appreciation of the complexity of the population. Expression of *OTX1*, *En1*, and *HoxA7* suggests the formation of cells characteristic of different CNS regions including midbrain and trunk[31]. As hypothesised earlier, this is likely to result, at least in part, from exposure to positional signalling from non-neural cell populations. Although the use of selective conditions results in a reduction in the complexity of the differentiation environment with ablation of many contaminating cell populations, initial formation of the neural lineage occurs as a consequence of unregulated pluripotent cell differentiation with concomitant formation of non-neural cell lineages including known sources of embryonic patterning information such as visceral endoderm[34].

## DIRECT DIFFERENTIATION FROM ES CELLS: EXPLOITATION OF THE DEFAULT DIFFERENTIATION PATHWAY?

The concept that neural fate is a default state for differentiating cells has emerged from experiments in *Xenopus* and mammals[39,40,41,42,43]. Culture of monolayer ES cells in chemically defined medium, in the absence of serum or exogenous factors, resulted in a rapid loss of pluripotence and an increase in neural specific gene expression but not mesoderm specific gene expression, supporting neural cell fate as a default for pluripotent cells[44]. This has been further exploited by Tropepe et al.[45] with the demonstration that culture of an ES cell suspension in chemically defined, serum-free medium supplemented with LIF results in formation of nestin positive sphere colonies. The frequency of sphere formation was low (~0.2%), suggesting that extensive cell selection had occurred, and although expression of mesodermal markers was not detected by RT-PCR, genes characteristic of early endoderm lineages were expressed. Neural precursor cells generated in this manner were not initially responsive to exogenous FGF2, nor did they express *Otx1*, a gene consistently expressed in the nestin positive cell populations generated by other approaches. Failure to express *Otx1* has been suggested to reflect the formation of a neural cell progenitor that is not anterior/posterior specified[45], however, expression of forebrain (*Emx2*) and hindbrain/spinal cord (*HoxB1*) markers was detected. Precursor cells within ES cell–

derived spheres were capable of further differentiation *in vitro* to both neural and glial lineages, in the apparent absence of non-neural cell types. Aggregation of single sphere colonies with embryonic morulae resulted in contribution to all tissues of the 9.5 d.p.c. embryo, suggesting a very broad differentiation potential.

## DIRECTED DIFFERENTIATION OF ES CELLS TO NEURAL PRECURSORS

The ability to direct differentiation of ES cells could be used to produce homogeneous populations of neural precursors in the absence of contaminating cell populations and potentially inappropriate signalling pathways. Two independent sources of potent neural inducing activities which direct the differentiation of ES cells to neural lineages have been identified[46,47].

In the first of these reports[46], adherent coculture of unaggregated ES cells with the stromal cell line PA6 resulted in efficient neural differentiation, with 92% of colonies expressing the neural marker NCAM, and <2% of colonies containing cells expressing mesodermal markers. Cells within individual colonies were heterogeneous, with the majority of cells expressing either β-tubulin type III (52%), a marker of neurons, or nestin (47%). Neural induction was shown to require a PA6-associated activity named stromal cell–derived neural inducing activity (SDIA), which was in part associated with the surface of the PA6 cells, with potential involvement of a soluble component. Although differentiation was characterised only on day 12, when terminal differentiation of precursors to neurons was apparent, the presence of nestin-positive cells suggested that differentiation had proceeded via the formation of a neural precursor population. Redirection of neural differentiation to a surface ectodermal cell fate in response to BMP4 suggested that neural determination occurred via a bipotential ectodermal intermediate, consistent with normal embryogenesis. However, the presence of both precursor and differentiated cells within individual colonies indicated that this differentiation was not synchronous. The potency of this cell population was potentially skewed, with very low levels of glial lineage formation (2%), and an overrepresentation of TH positive neurons within the β-tubulin type III expressing population (30%). Cell populations enriched in TH positive neurons after culture for 12 days on PA6 cells were implanted into the striatum of rats treated with 6-hydroxydopamine (6-OHDA), which selectively ablates TH positive, dopaminergic neurons within the nigro-striatal region. These implants restored TH positive regions around the site of the graft, demonstrating the survival and integration of these cells *in vivo*. While the expression of positional markers within SDIA-induced neural precursors was not addressed, the low level of non-neural cell types that accompany differentiation suggests that endogenous signalling between heterogeneous cell types is likely to be minimal. However the presence of additional PA6-derived signals cannot be excluded.

Directed differentiation of ES cells to neural lineages has also been achieved by culture of ES cell aggregates in conditioned medium (MEDII) from the human hepatocellular carcinoma cell line HepG2. Originally identified by the ability to differentiate ES cells in adherent culture to early primitive ectoderm–like (EPL) cells[14,48], MEDII has been recently shown to direct the differentiation of EPL cell aggregates formed from aggregated ES cells to a neural precursor population[47]. By day 9, aggregates formed and differentiated in MEDII have adopted a distinctive morphology, comprising convoluted, columnar, stratified epithelium reminiscent of the embryonic neural plate/neural tube. This is a distinctive cell morphology not reported for other differentiation systems. The homogeneity of pluripotent cell differentiation in response to MEDII is reflected in this uniform morphology, widespread expression of the neural cell adhesion molecule N-CAM (95.7%) by the population, and by the inability to detect progenitor or differentiated mesoderm and extraembryonic endoderm cell populations by morphology or gene expression at any stage during differentiation[47].

Neural precursors formed after 9 days in this system express *Sox1*, *Sox2*, nestin, and N-CAM and exhibit a broad differentiation potential with the ability to form neurons, glial lineages, and neural crest, the latter not previously observed from ES cell–derived precursors. Differentiation to glia and neural crest was achieved in response to EGF/FGF2/PDGF[32] and staurosporine[49], respectively, and resulted in formation of effectively homogeneous differentiated cell populations. Neural crest formation was determined by both morphological criteria and expression of the neural crest marker *Sox10*. Alternate homogeneous differentiation of these cells in response to different exogenous factors implies homogeneity of the starting population and an absence of competing differentiation inducing signals within the differentiation environment. Furthermore, neural crest formation in response to staurosporine is a property shared with the neural tube of the quail embryo, aligning the biological properties of the *in vivo* and *in vitro* cell populations.

Differentiation in response to MEDII follows closely establishment of the neural lineage in the embryo, with the sequential formation of primitive ectoderm, neural plate, and neural tube equivalent cell populations, demonstrated by the temporal and sequential regulation of genetic markers for each of these populations. For example, the transient up regulation in *SOX1* expressing cells of *GBX2*, a homeobox gene expressed by neural plate and down regulated on neural tube closure, during differentiation in response to MEDII suggests the sequential formation of neural plate and neural tubelike populations. Analysis of positionally restricted genes suggested that this cell population had not up regulated genes characteristic of dorsal/ventral specification or genes indicative of the forebrain, hindbrain, or trunk. This has been interpreted to suggest alignment with an early midbrain cell population, or alternatively a naive, or unpatterned, neuroepithelium. This assignment is consistent with the observed lack of contaminating cell populations and, hence, inherent signalling within the aggregates[16].

## THERE IS MORE THAN ONE WAY TO SKIN A CAT

It is apparent from the recent literature reviewed here that a number of divergent approaches can be used to generate a nestin-positive, neural precursor or progenitor population from ES cells in culture. However, although nestin expression is seen within the neural plate at 8.5 d.p.c., expression persists within the proliferating progenitor cells throughout CNS development, thus expression of nestin does not necessarily align cells with a specific embryonic precursor cell population[50]. Similarly, although on the face of it the cell populations generated appear relatively similar, the lack of systematic characterisation does not allow comparative assessment of cell identity or developmental plasticity. The impact of contaminating cell populations, endogenous signalling pathways, and acquisition of positional specification has yet to be appreciated or experimentally assessed, although preliminary evidence suggests that the developmental potency of cell populations generated by alternative methodologies may differ.

Populations of neural progenitors formed from ES cells *in vitro* have projected applications in research, as a model system for understanding the molecular and cellular processes of cell fate determination in establishment and elaboration of the neural cell lineage, and in medicine, as a system for the production of implantable cell populations with therapeutic applications. The considerations of cell generation for each system may be different. Research models would be best served by formation of cell populations in a manner that recapitulates embryogenesis, with faithful representation of intermediate cell populations and induction and patterning events. The methodologies used to achieve production of implantable populations may be less important, but rigorous regulatory demands are likely to be placed on reproducibility, homogeneity, and authenticity. Both requirements would be met by generation of the lineage *in vitro* in a manner that closely reiterates embryogenesis, with the sequential and temporal elaboration of progressively more differentiated intermediate cell populations. Differentiation would ideally be relatively synchronous, such that cell populations enriched in intermediate cell types could be captured, characterised, and manipulated. Homogeneity throughout the differentiation process is likely to be preferable to selection-based systems as it minimises the potentially deleterious effects of inappropriate signalling, which may include adverse effects on cell identity and potential. Although these aspirations might appear ambitious, rapid recent advance in divergent relevant areas such as embryogenesis, cell biology, and stem cell therapy provides confidence that the intellectual and technical underpinning required for success will be achievable.

## ACKNOWLEDGEMENTS

The authors would like to acknowledge support from the Australian Research Council and the Cell Therapy Program, Bresagen. We would like to make special mention of the contributions of Dr. Paul Tolstoshev.
